# Building Healthier Communities in the Workplace: The Impact of a Year-Long Lifestyle Intervention on Food Access, Quality of Life, and Health Metrics

**DOI:** 10.3390/nu17040637

**Published:** 2025-02-11

**Authors:** Orit Afumado Yona, Mona Boaz, Shir Ben-Yaish, Vered Kaufman-Shriqui

**Affiliations:** 1 Department of Nutrition Sciences, School of Health Sciences, Ariel University, Ariel 4076405, Israel; orit.yona@moh.gov.il (O.A.Y.); monabo@ariel.ac.il (M.B.); shir.ben-yaish@moh.gov.il (S.B.-Y.); 2 Division of Nutrition, Ministry of Health, Jeremiah 39, Jerusalem 9446724, Israel; 3 Israel Center for Disease Control, Israel Ministry of Health, Ramat Gan 5262100, Israel

**Keywords:** community engagement, factory workers, food menu, financial cost, physical activity, registered dietitian

## Abstract

Background/Objectives: The present study assessed the impact of a year-long community-engaged lifestyle intervention to improve healthy food access, anthropometric measures, and quality of life among factory workers. Methods: A total of 80 workers aged 20–65 participated in this quasi-experimental pre–post intervention with nine dietitian-led sessions, weekly physical activity classes, and adjustments to the factory food menu. Volunteer health leaders from the workforce played a pivotal role as project stakeholders, promoting the initiative, liaising with the food supplier, advocating for physical activity hours with management, and supporting activity dissemination. Data were collected at baseline, four months, and one year, including weight, waist circumference, dietary intake, physical activity, and quality of life (SF-36 questionnaire). Results: Waist circumference significantly decreased after 4 and 12 months. Regression models showed that each additional year of age correlated with a 0.72 cm reduction, while over 150 min of weekly physical activity was associated with a 6.58 cm decrease. The mental health component of the quality of life scores improved from 73.1 ± 18.5 to 78.7 ± 17.6 (*p* = 0.017), alongside reductions in sugar and sodium intake. The intervention cost ILS 4875 (EUR 1314 or USD 1369) per employee annually. Conclusions: This community-engaged, dietitian-led intervention significantly improved factory workers’ health and well-being, providing a cost-effective solution.

## 1. Introduction

A sedentary lifestyle and increased consumption of high-energy foods are associated with obesity. In addition to these factors, the factory work environment is associated with obesity due to long work hours, a lack of access to nutritious food, and limited access to or a lack of time for physical activity [1,2]. Obesity serves as an independent risk factor and a mediating factor for the development of metabolic diseases, depression, and mortality. Adults spend approximately one-third of the day in the workplace, making it a unique framework for testing the efficacy of healthy lifestyle strategies [3,4]. Furthermore, obesity-related health conditions result in substantial economic and productivity losses, emphasizing the urgency for workplace-based interventions [5].

Unhealthy food environments, including high-fat meals served in the workplace, are associated with obesity. These meals provide few vegetables and fruit but often include high-fat and sugar-rich desserts. There are limited food options that meet the guidelines for healthy nutrition. In addition, ultra-processed and energy-rich foods and beverages, such as sweets, snacks, and pastries, are offered to workers in vending machines [6] and in meetings and conferences [7].

Evidence suggests that participatory approaches, where workers are engaged in designing and implementing interventions, can lead to greater program acceptance, sustainability, and behavior change. For instance, community-based participatory interventions have been shown to improve the work–life balance of caregivers providing informal care. Another study found that empowering workers to identify barriers and propose context-specific solutions helped to improve public health intervention efficacy [8,9]. These participatory strategies align with recent trends in public health research, highlighting their potential to foster long-term behavior change and ensure intervention effectiveness [10].

Studies indicate that fostering collaboration in meal planning, food procurement, and menu adjustments can significantly improve dietary quality and reduce unhealthy food consumption in workplace settings [11]. However, further investigation is needed to delineate the optimal characteristics of such collaborations, including the role of leadership, communication strategies, and long-term engagement [12,13].

This study evaluates the efficacy of a dietitian-led, community-engaged lifestyle intervention conducted among factory workers, with volunteer health leaders serving as stakeholders to drive program implementation and sustainability.

## 2. Materials and Methods

### 2.1. Design

A quasi-experiment, this self-controlled clinical trial was conducted over one year. Participants were recruited from among factory workers aged 20–65 who had six months or more of employment at the participating factory. The composite lifestyle intervention included individual and group meetings with a registered dietitian (RD), lectures, physical activity classes, and changes to the food menu served at the factory. The inclusion criteria included being a full-time factory employee aged 20–65 years, employed for at least six months before the study. The exclusion criteria included employees on extended leave or planning to leave within six months, employees with medical conditions preventing participation in physical activity, and individuals undergoing significant dietary interventions unrelated to the study.

Pre–post data collection included anthropometrics (weight, BMI, and waist circumference); nutritional intake assessments (validated dietary questionnaires); physical activity tracking (weekly minutes of activity); quality of life (SF-36 scores); and absenteeism records. Data were collected via in-person interviews conducted at baseline, four months, and one year from the study onset.

### 2.2. Community Engagement

Community engagement was a central component of the intervention. This intervention was guided by behavioral change models, mainly the social–cognitive theory [14], which explains behavior change as a result of reciprocal interactions between a person’s behavior, cognition, and environmental influences, including team processes and workplace health promotion, recognizing that employee involvement is crucial for the success and sustainability of workplace health initiatives. Prior to implementation, a stakeholder committee was formed, consisting of a kitchen worker, two administrative staff members, one financial staff member, and the chief executive officer. The committee collaborated with two senior RDs to design the intervention through four planning meetings, during which, the responsibilities and schedules for participation in nutrition and physical activity meetings were defined. Monthly feedback sessions ensured that worker concerns were addressed, and adjustments were made accordingly.

Workers were directly involved in selecting physical activity schedules, negotiating menu adjustments with the food supplier, and advocating for workplace policy changes (e.g., extended break times for activity).

An open meeting was held, inviting all workers to identify key health challenges and propose solutions. Sedentary behavior and the poor quality of lunches provided by the factory were identified as primary concerns across departments, including drivers, administrative workers, and finance personnel. Workers also requested ergonomic improvements, such as better chairs and tables. Through negotiations with management, agreements were reached to fund after-work physical activity lessons, revise the food supplier to improve meal quality, and conduct nutrition workshops during work hours. Monthly follow-up meetings were held with the stakeholder committee to monitor and adjust the program as needed.

### 2.3. Intervention Program

The intervention program included three personal counseling sessions with a dietitian, six group meetings led by a dietitian, four lectures by a dietitian to increase awareness of a healthy lifestyle in general and healthy Mediterranean nutrition in particular, and a lecture by a certified physical fitness instructor on the importance of physical activity.

The three individual counseling sessions with a dietitian comprised the core of the intervention, occurring at baseline, after 4 months and 12 months from the start of the intervention program. The six group sessions led by a dietitian were held once every six weeks during the 4–12-month period. Regarding the four lectures by a dietitian to raise awareness of a healthy lifestyle in general and a healthy Mediterranean diet in particular, two lectures occurred during the 0–4-month period and two additional lectures, one every four months, occurred during the 4–12 month period. A lecture by a certified fitness instructor on the importance of physical activity was held during the 4–12-month period. 

The in-person nutrition counseling meetings were designed to help participants make healthier dietary decisions in the workplace and at home, strongly emphasizing adherence to Mediterranean dietary guidelines. The participants were guided in analyzing the factory menu to make informed meal selections, practice portion control, and incorporate more whole foods, vegetables, healthy fats, and lean proteins into their diet. Personalized goal-setting strategies were introduced to help individuals align their eating habits with Mediterranean principles, focusing on meal planning, nutrient balance, and reducing high-sugar and high-sodium intake (e.g., replace mayonnaise-based salad dressing with olive oil and lemon). Additionally, the sessions addressed individual dietary challenges, including barriers to healthy eating, managing hunger at work, emotional eating, and sustaining motivation. The participants were empowered to make sustainable, long-term changes that promoted overall well-being by integrating practical goal-setting and adherence strategies. A detailed description of the intervention program can be found in Table A1.

### 2.4. Anthropometric Measures

Weight and height were measured using the Tanita scales model BC 418 MA. Waist circumference was measured using a flexible measuring tape wrapped around the body, with the subject standing with legs parallel, shoulder-width apart and their hands comfortably at the sides of the body. The measurement location was at the height of the umbilicus. All measurements were performed twice, and the average value of the two measurements was recorded.

### 2.5. Food Intake

The study participants were administered 24 h food recalls by registered dietitians, using the MABAT (National Health and Nutrition Surveys) survey questionnaire, used regularly by the Israel National Center for Disease Control’s (NCDC) survey unit.

Macro- and micronutrient intakes were calculated. The nutrition analysis was performed using the “Tzamert” software 1.0.1 (Israel Ministry of Health) [15]. The food items were analyzed for macronutrients (protein, fat, and carbohydrates) in grams, and the amount of energy (in kilocalories) was calculated. In addition, the intake of the following nutrients was calculated: monounsaturated fatty acids, polyunsaturated fatty acids, saturated fat, sodium, and sugars (including the mono- and di-sugars found in food and added to it, such as glucose, sucrose and fructose, and galactose).

### 2.6. Health-Related Quality of Life

The relationship between employee well-being and workplace performance has been well-documented. Poor mental and physical health has been linked to cardiovascular disease, anxiety, depression, absenteeism, reduced productivity, and a higher staff turnover [16]. To assess the intervention’s impact on quality of life indicators, the SF-36 questionnaire was used [17]. This concise, multidimensional tool evaluates health-related quality of life across the following eight components: physical functioning, physical role limitations, bodily pain, general health, vitality, social functioning, mental role limitations, and mental health. These components combine into two indices, Physical Component Summary (PCS) and Mental Component Summary (MCS) [18]. SF-36 scoring involves two steps, as follows: responses are recorded on a 0–100 scale, followed by averaging the items within each component to calculate the eight dimensions and two summary indices [19].

In addition, work absenteeism was assessed using the Health and Work Questionnaire of the World Health Organization [20].

### 2.7. Physical Activity

Data on physical activity type and duration were obtained during an in-person interview with the participants at baseline, 4 months, and 12 months from baseline using the MABAT National Health Questionnaire [21]. For further analysis, we classified the participants according to the international guidelines for performing 150 min of physical activity weekly.

### 2.8. Statistical Analysis

The data analysis was conducted using SPSS v26 (IBM Inc., Armonk, NY, USA). Missing values were addressed by reviewing the original data sources. Continuous variables were assessed for normality using the Kolmogorov–Smirnov test. Normally distributed variables are presented as mean ± standard deviation, while non-normal variables are described as median and range (min–max). Pre- and post-intervention comparisons of continuous variables were performed using paired *t*-tests or Wilcoxon Signed Ranks tests, as appropriate. Nominal variables are described as n (%) and were analyzed using McNemar’s test. To examine changes in outcome measures over time and the interaction between sex and treatment duration, an analysis was performed that included all employees who began the intervention (intention-to-treat analysis). A generalized linear mixed model (GLMM) with a random effect was applied to evaluate the program’s overall effectiveness. Our analysis accounted for several control variables to ensure the validity of our findings. Age and sex were included as covariates in multiple regression models. Baseline physical activity levels were considered to assess relative improvements. Household income and marital status were analyzed as potential factors influencing participation. Smoking status was included in the dietary and physical activity evaluations.

Separate models were fitted for each outcome variable. The linear outcome variables were weight, waist circumference, SF-36 questionnaire score, number of minutes of physical activity, satisfaction with food, and the number of days absent from work. The significance level was set at *p* < 0.05. All tests were two-sided.

## 3. Results

**Figure 1:**
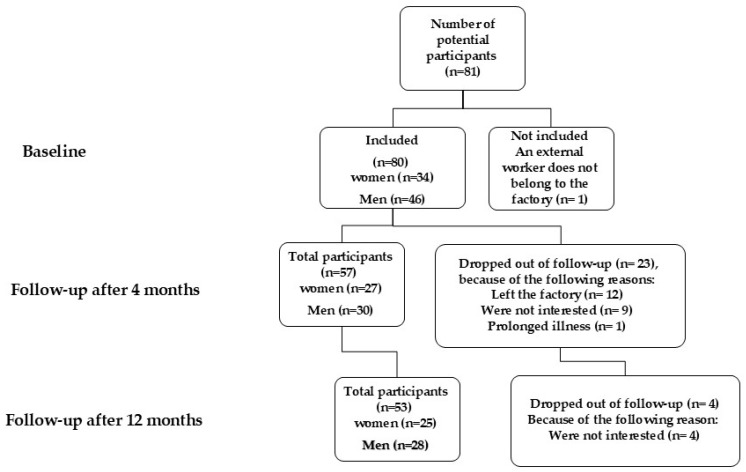
Flow chart of study participants.

A total of 81 participants were recruited, of whom 34 were women. After 4 months, 57 workers (71%) remained in the study, as 12 had discontinued employment at the factory and 9 people were not interested in continuing the intervention program. One worker discontinued participation for medical reasons, and another retired earlier than expected. After 12 months, 53 (66%) participants remained in the study (Figure 1).

As presented in Table 1, at baseline, the sample included slightly more men than women, with the majority of participants being married. Across quality-of-life measures (SF-36), the participants scored relatively high in physical functioning and social functioning, while their vitality and mental health scores were comparatively lower. Men generally reported slightly better scores across most quality of life domains compared to women. Physical activity levels varied widely, with men showing higher average weekly minutes than women among those who reported activity.

The physical activity results for the entire group at 4 and 12 months were as follows: after 4 months, there was a non-significant increase in the number of weekly physical activity minutes compared to baseline (*p* = 0.751). However, there was a significant increase in the percentage change in weekly physical activity minutes (*p* = 0.033). After 12 months, a significant increase in weekly physical activity minutes was observed compared to baseline (mean ± SE: 390.9 ± 65.9 vs. 259.2 ± 37.8, *p* = 0.039). The percentage change in weekly physical activity minutes was also significant (86.2 ± 33.1, *p* = 0.033).

In a multiple linear regression analysis, weight at four months was positively associated with age, with each additional year corresponding to a 0.26 kg increase, independent of sex. Participants from households with a net monthly income exceeding ILS 10,000 had a significantly higher weight of 16.46 kg (SE = 3.09, *p* < 0.001). Similarly, participants attending healthy lifestyle lectures or groups more than twice had a higher weight by 7.01 kg (SE = 3.01, *p* = 0.02) than those who did not. While total minutes of daily physical activity during the 12-month intervention showed a marginal association with a lower weight (B = −0.008, SE = 0.004, *p* = 0.055), this effect did not reach full statistical significance.

Waist circumference decreased significantly after four months, with reductions of −2.7 ± 6.6 cm (*p* = 0.002) in women and −5.8 ± 12.3 cm (*p* = 0.006) in men. These improvements persisted at 12 months, with reductions of −5.5 ± 3.6 cm (*p* < 0.001) in women and −4.3 ± 3.8 cm (*p* < 0.001) in men compared to baseline.

Regression analysis showed that each additional year of age was associated with a 0.72 cm increase in waist circumference, regardless of sex, while exercising at least 150 min per week was significantly and inversely associated with waist circumference.

The findings of the multiple linear regression analysis with a random effect of the change in waist circumference are presented in Table 2. A significant reduction in waist circumference was observed at both 4 months (−4.24 ± 1.07 cm, *p* < 0.001) and 12 months (−4.34 ± 1.22 cm, *p* < 0.001) compared to baseline, after adjusting for sex, age, and physical activity. Each additional year of age was associated with a 0.72 ± 0.11 cm increase in waist circumference (*p* < 0.001). Physical activity of more than 150 min per day was associated with a 6.58 ± 3.39 cm reduction in waist circumference (*p* = 0.05).

At 12 months, both vitality and general mental health had increased significantly from baseline. These findings were robust even after controlling for age and sex.

The multiple linear regression analysis with a random effect (Table 3) showed a significant increase in the mental health component score (SF-36) at 12 months compared to baseline, with a mean improvement of 4.43 ± 2.32 points (*p* = 0.05), adjusted for sex and age. Sex and age were not significantly associated with changes in mental health scores, although there was a non-significant trend suggesting lower scores among women (−6.88 ± 3.77, *p* = 0.07).

The total physical activity levels did not significantly increase during the intervention. However, married workers engaged in physical activity for less than half the time that single workers did (*p* < 0.001), irrespective of age or sex.

**Figure 2:**
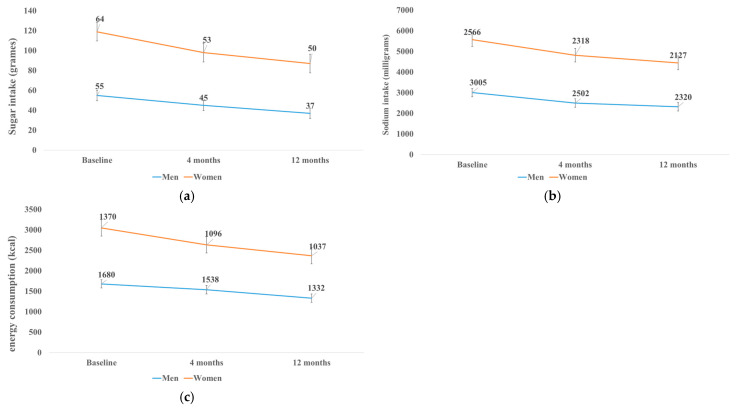
Changes in nutrients per day after 4 and 12 months compared to the baseline of the intervention (**a**) changes in sugar intake by gender after 4 and 12 months from the baseline of the intervention; (**b**) changes in sodium intake by gender after 4 and 12 months from the baseline of the intervention; and (**c**) changes in energy intake by gender after 4 and 12 months from the baseline of the intervention.

As shown in Panel A of Figure 2, a significant reduction in sugar intake was observed over the intervention period. Men reduced their sugar intake from 55 g/day at baseline to 45 g/day at 4 months and 37 g/day at 12 months, while women decreased their intake from 64 g/day to 53 g/day and 50 g/day at the same time points, with a mean reduction across the population of 18.55 ± 5.93 g/day (*p* < 0.01). Panel B illustrates significant declines in sodium intake, with an overall reduction of 484.78 ± 202.43 mg/day (*p* < 0.01). Men reduced their sodium intake from 3005 mg/day at baseline to 2502 mg/day at 4 months and 2320 mg/day at 12 months, while women decreased their intake from 2566 mg/day to 2318 mg/day at 4 months and 2127 mg/day at 12 months. Sodium intake at 12 months was significantly lower in women compared to men by −765.01 ± 189.21 mg/day (*p* < 0.001). Panel C shows a significant decline in energy intake for both sexes, with men reducing their intake from 1680 kcal/day at baseline to 1538 kcal/day at 4 months and 1332 kcal/day at 12 months, and women from 1370 kcal/day to 1096 kcal/day and 1037 kcal/day (*p* < 0.05). Despite these improvements, cholesterol intake remained within recommended ranges, while fruit and vegetable consumption showed no significant change, and dietary fiber and essential fatty acid intake continued to fall 30–60% and 8–37% below recommendations, respectively (Table 4). Improvements in the factory dining room menu contributed to these results, with a significant reduction in the perceived unhealthiness of meals by 12 months, reflecting a lower fat and salt content, underscoring the intervention’s success in promoting healthier dietary habits.

At the 12-month follow-up, employee absenteeism had declined significantly compared to baseline. Our findings showed a significant reduction in absenteeism over 12 months, with a 63.6% decrease from baseline (mean ± SD days were at baseline 1.1 ± 3.8 and decreased to 0.4 ± 1.8 days, *p* = 0.0002).

The total financial cost of the intervention program was ILS 4875 (EUR 1273 and USD 1358) per participating employee. Additionally, the employer’s annual cost for employee absence due to participation in the intervention was ILS 8299 (EUR 2161 and USD 2311). The overall cost of implementing the program, including all components, amounted to ILS 389,967 (EUR 101,819 and USD 108,728), averaging ILS 4875 (EUR 1273 and USD 1358) per employee.

## 4. Discussion

The findings of the present study demonstrate the efficacy of a community-engaged, dietitian-led workplace intervention in improving health and well-being among factory workers. Significant reductions in waist circumference, sugar intake, and sodium consumption were observed, alongside improvements in mental health scores and employee perceptions of workplace nutrition. Although weight and BMI changes were less pronounced, trends toward improvement were evident. The intervention also reduced employee absenteeism, emphasizing its potential economic benefits. These results suggest that participatory workplace health programs can address key risk factors for chronic diseases while promoting healthier food environments and fostering employee well-being.

The observed reduction in waist circumference, which persisted through the 12-month intervention, aligns with prior studies indicating the efficacy of workplace-based health promotion programs in reducing central obesity [21]. This finding is particularly important, as abdominal obesity is a major risk factor for metabolic and cardiovascular diseases. The observed association between physical activity and waist circumference reductions corroborates existing research, highlighting the role of regular exercise in managing body composition [22]. However, the lack of significant weight loss in men and the modest improvements in BMI may reflect variations in individual responses and engagement levels and the challenges of achieving weight loss in workplace settings [22]. Intervention programs to improve nutrition and reduce obesity require participants to have a high involvement and personal commitment. However, such interventions also require collaboration, implementation resources, and encouragement from management [23].

The improvements in dietary behaviors, particularly the reduced sugar and sodium intake, further underscore the impact of workplace interventions in shaping healthier eating patterns. Excessive sugar and sodium intake are well-documented contributors to obesity, hypertension, and cardiovascular disease [24,25].

Overall, the results of the present study are consistent with other studies that have demonstrated the efficacy of dietary changes in workplace settings, especially when such interventions include menu modifications and active employee involvement [3,26]. Notably, the Mediterranean-style changes to the factory dining menu played a pivotal role in reducing the consumption of unhealthy foods, aligning with evidence that improving food availability and quality can significantly influence dietary habits [27].

In addition to the physical health benefits, the intervention yielded meaningful improvements in mental health scores, as measured by the SF-36. These results align with findings from other workplace interventions that have emphasized the link between improved nutrition, physical activity, and mental well-being [28]. The participatory approach of engaging workers in the program’s design and implementation likely contributed to these mental health gains, as prior research suggests that active involvement enhances program acceptance and fosters a sense of empowerment [29]. Nevertheless, the lower engagement observed among married workers and lower-income employees highlights the need for tailored strategies to address participation barriers, such as providing additional incentives or flexible schedules [27].

This study contributes to the growing body of evidence supporting community-engaged workplace interventions as a cost-effective strategy for improving employee health and well-being. The reductions in absenteeism and perceived improvements in workplace food quality underscore the potential economic and productivity benefits for employers. One of the options offered by this study to facilitate employee choices is an environmental and dietary change under a dietitian’s ongoing and consistent guidance. A recent survey conducted in Poland among factory workers showed that people with obesity placed the highest value on healthy food options [30]. People with a normal BMI were more responsive to healthy food subsidies, nutritional advice provided by a dietitian, and access to fitness facilities [19]. Similar studies using peer-support models and worker engagement strategies have demonstrated a greater intervention effectiveness due to increased motivation and accountability. While the presence of volunteer leaders may influence engagement, this is an inherent strength in workplace-based interventions, rather than a methodological flaw. Future studies may focus on identifying worker populations that can benefit more from workplace nutrition consultation.

This study stands out for its innovative, community-engaged approach to workplace health promotion, where workers played a central role in designing and implementing the intervention. Unlike traditional top-down programs, this participatory model ensured that the intervention addressed employee-identified priorities, such as sedentary behavior and poor meal quality. Integrating Mediterranean dietary principles into the factory menu, guided by worker input, represents a novel strategy for improving food access and promoting healthier eating patterns in industrial settings. Additionally, the program’s comprehensive design—combining personalized dietitian consultations, group sessions, and environmental changes—addresses multiple dimensions of health, making it a robust model for tackling the obesogenic workplace environment.

However, the lack of a comparison group limits causal inferences, and the small sample size and relatively high dropout rate reduced the statistical power for specific outcomes, such as fruit and vegetable intake. While the absence of a control group is a limitation, our study provides valuable real-world evidence for the effectiveness of a dietitian-led workplace health intervention. Additionally, heterogeneity in baseline characteristics and limited engagement among low-income workers posed challenges. Although randomized controlled trials (RCTs) provide the gold standard for causal inference, they may not always be practical or ethical in workplace settings. Given the importance of equitable health access, withholding a potentially beneficial intervention from a control group can be ethically complex. Future studies should explore quasi-experimental designs (e.g., matched control groups or stepped-wedge trials) to improve causal interpretation [30].

Despite these limitations, the study contributes valuable evidence on participatory interventions and highlights their feasibility in industrial settings. Real-world workplace interventions often involve small samples. Workplace-based health interventions frequently target specific employee groups, limiting sample sizes due to organizational constraints, participation rates, and feasibility considerations. Despite the relatively small sample, our study provides valuable, real-world evidence applicable to similar work environments. Additionally, the longitudinal design (baseline, 4-month, and 12-month follow-ups) strengthens the reliability of our findings. The observed consistent trends in dietary changes, waist circumference reduction, and mental health improvements suggest meaningful intervention effects, even in a smaller cohort.

Future research should focus on enhancing engagement strategies for underserved populations and expanding these interventions across diverse workplace settings. Additionally, extending follow-up assessments to two or more years will help to determine the long-term sustainability of health improvements. Incorporating job satisfaction measures in future studies would also provide a more holistic evaluation of workplace interventions, capturing health and well-being outcomes. Future studies could include a formal cost–benefit analysis, healthcare utilization data (e.g., reduced doctor visits and medication costs), and absenteeism and presenteeism analysis to estimate productivity gains and employer healthcare savings projections based on risk reduction models.

Lastly, while our study primarily focused on quantitative outcomes, we recognize the added value that participant narratives and subjective experiences could bring in understanding the intervention’s real-world impact.

## 5. Conclusions

The findings of this study highlight the potential of integrating a community-engaged, dietitian-led intervention program into the workplace for health promotion to improve dietary habits, health outcomes, and employee well-being. Expanding this approach through the introduction of a full-time nutritionist in the workplace could yield even greater benefits by consistently addressing the obesogenic environment. A nutritionist could provide ongoing guidance to improve menu planning with food suppliers, offer healthy options for employees bringing food from home, and ensure the availability of nutritious refreshments at workplace events. Continuous engagement with employees would also foster the assimilation of health messages, promote a culture of health consciousness, and sustain long-term behavior change.

From a broader economic perspective, the implementation of health promotion programs warrants cost–benefit analyses to evaluate their impact on labor productivity, absenteeism, and overall health expenditures. This study demonstrates the feasibility and value of workplace interventions, suggesting the need for state-level financial support to encourage the broader adoption of such programs. By incorporating dietitians and fitness trainers into workplace health promotion strategies, policymakers and employers can play a critical role in reducing chronic disease risk factors, improving workforce health, and enhancing productivity. These findings offer a framework for scaling such interventions across diverse workplaces, contributing to public health and economic sustainability.

## Figures and Tables

**Table 1:** Baseline characteristics of study participants.

Variable	All n = 80	Women n = 34	Men n = 46
Sex n (%)		(42.5) 34	46 (57.5)
Marital Status n (%)			
Single	24 (30)	(11.8) 4	(43.5) 20
Married	(57.5) 46	(64.7) 22	24 (52.2)
Divorcee	(10.0) 8	(20.6) 7	(2.2) 1
Widowed	2 (2.5)	(2.9) 1	(2.2) 1
Weight (Kg) (Mean ± SD)	77.7 ± 18.9	72.9 ± 19.5	81.2 ± 17.8
Waist circumference (cm) (Mean ± SD)	93.1 ± 15.7	88.2 ± 14.9	96.73 ± 15.5
Average number of minutes of physical activity per week (Mean ± SD)	268.4 ± 191.4	172.5 ± 92.2	320.8 ± 212.1
SF-36 dimensions score (Mean ± SD)			
PF = Physical Functioning	92.6 ± 21.0	90.0 ± 29.8	94.7 ± 9.2
RP = Role Physical	81.1 ± 32.5	81.0 ± 33.2	81.2 ± 32.4
RE = Role Emotional	80.5 ± 34.8	75.9 ± 41.7	84.2 ± 28.1
VT = Vitality (Energy and Fatigue)	59.8 ± 21.1	59.8 ± 19.7	59.8 ± 22.5
MH = Mental Health	72.4 ± 18.7	71.3 ± 19.2	73.3 ± 18.5
SF = Social Functioning	85.4 ± 24.0	85.3 ± 21.1	85.4 ± 26.5
BP = Bodily Pain	76.6 ± 26.2	75.1 ± 26.0	77.9 ± 26.6
GH = General Health	74.7 ± 19.9	72.1 ± 23.7	76.8 ± 16.3

After four months, women showed a significant reduction in body weight (−1.71 ± 3.31 kg, *p* = 0.021), corresponding to a BMI decrease of −0.68 ± 1.35 kg/m^2^ (*p* = 0.025). No significant changes in body weight were observed among men. By 12 months, no differences in weight or BMI were detected compared to baseline, although weight at four months was significantly lower than at 12 months (*p* = 0.003).

**Table 2:** Changes in waist circumference and associated factors over 12 months (GLMM analysis).

Variable	Model 1			Model 2		
	B	SE	*p*-Value	B	SE	*p*-Value
Baseline (time 0)	ref			ref		
4 months	−5.08	1.66	0.02	−4.24	1.07	<0.001
12 months	−4.06	1.65	0.01	−4.34	1.22	<0.001
Sex						
female				−20.38	3.63	<0.001
Age (years)				0.72	0.11	<0.001
Physical activity over 150 min a day				−6.58	3.39	0.05
Constant	93.08	1.80	<0.001	75.73	5.04	<0.001

Random effect multiple linear regression analysis (GLMM—Generalized Linear Mixed Model), standardized *p* value for multiple comparisons.

**Table 3:** Changes in mental health (SF-36) score regression among the sample population after 12 months compared to the baseline of the intervention (GLMM Analysis).

Variable	Model 1			Model 2		
	B	SE	*p*-Value	B	SE	*p*-Value
Constant	74.81	2.33	<0.001	66.27	8.53	<0.001
Baseline (time 0)	ref			ref		
12 months	4.39	2.37	0.06	4.43	2.32	0.05
Sex						
Female				−6.88	3.77	0.07
Age				0.29	0.18	0.10

Random effect multiple linear regression analysis (GLMM—Generalized Linear Mixed Model), standardized *p* value for multiple comparisons.

**Table 4:** Changes in selected nutrients over time: baseline, 4 months, and 12 months.

Nutrient/Category	Baseline Intake (Mean ± SE)	After 4 Months (Mean ± SE)	After 12 Months (Mean ± SE)	*p*-Value (Baseline vs. 12 Months)
Total Energy Intake	1985 ± 210 kcal/day	1923 ± 195 kcal/day	1892 ± 182 kcal/day	0.089
Protein Intake	78.2 ± 5.4 g/day	75.8 ± 5.1 g/day	74.2 ± 4.9 g/day	0.075
Fat Intake	82.6 ± 7.1 g/day	79.3 ± 6.8 g/day	77.9 ± 6.5 g/day	0.081
Carbohydrate Intake	250.4 ± 18.2 g/day	245.1 ± 17.5 g/day	240.3 ± 16.8 g/day	0.094
Cholesterol Intake	289.9 ± 34.3 mg/day	240.3 ± 28.6 mg/day	238.6 ± 24.3 mg/day	0.080
Dietary Fiber Intake	10.2 ± 1.5 g/day	11.5 ± 1.3 g/day	11.8 ± 1.2 g/day	0.078
Essential Fatty Acids	3.4 ± 0.6 g/day	3.8 ± 0.5 g/day	3.9 ± 0.5 g/day	0.062
Fruit and Vegetable Intake	285.6 ± 25.3 g/day	292.1 ± 24.8 g/day	290.8 ± 24.1 g/day	0.115
Sugar Intake	58.8 ± 4.9 g/day	47.1 ± 4.2 g/day	42.4 ± 3.7 g/day	0.002
Sodium Intake	2817.3 ± 223.6 mg/day	2501.2 ± 195.8 mg/day	2237.4 ± 148.3 mg/day	0.026

## Data Availability

The original contributions presented in this study are included in the article. Further inquiries can be directed to the corresponding author.

## Abbreviations

The following abbreviations are used in this manuscript:

BMI	Body Mass Index
CDC	Center for Disease Control
GLMM	Generalized Linear Mixed Model
PA	Physical Activity
RCT	Randomized Controlled Trial
SD	Standard Deviation
SE	Standard Error
WHO	World Health Organization

## Appendix A

**Table A1:** Description of the intervention program.

Study Stage	Type of Intervention	Frequency	Number of Sessions	Purpose of the Intervention
0–4 months	Personal nutritional counseling by a dietitian	Twice during the first stage	Two sessions	According to medical history and blood tests, each employee has defined goals of nutritional consumption and eating behavior, physical activity, the goal of weight loss, and guidance for a healthy lifestyle in general and healthy nutrition in particular
0–4 months	Lectures to raise awareness about a healthy lifestyle in general and healthy nutrition in particular	Once a month	Two sessions	Lectures led by a dietitian
4–12 months	Personal nutrition counseling by a dietitian	Once during the second stage	One session	Each employee has scheduled a follow-up appointment with the goal of weight loss and guidance for a healthy lifestyle in general and healthy nutrition in particular (as decided in the first meetings)
4–12 months	Lectures on a healthy lifestyle, healthy nutrition, and physical activity	Once every 4 months	Two sessions	Lecture by a dietitian and a lecture by a fitness trainer
4–12 months	Group meeting led by a dietitian	Once a month	Six sessions	Group meeting led by a dietitian on healthy eating behaviors, a healthy lifestyle, and healthy nutrition
The entire study period	Exercise during the work organized in collaboration with factory management	Twice a week in separate groups for men and women	Twice a week	Aerobic exercise and strength exercises under the guidance of a fitness trainer
The entire study period	Improving the food served in the factory at lunch under the principles of the Mediterranean diet and monitoring meeting the standards of this diet	Checking the menu each week before arriving at the factory dining room	Once a week	Replacing the food supplier and supervising the dietitian on the composition and variety of foods served in the dining room under the principles of the Mediterranean diet
